# Intrinsic brain abnormalities of irritable bowel syndrome with diarrhea: a preliminary resting-state functional magnetic resonance imaging study

**DOI:** 10.1186/s12880-020-00541-9

**Published:** 2021-01-06

**Authors:** Weiqun Ao, Yougen Cheng, Mingxian Chen, Fuquan Wei, Guangzhao Yang, Yongyu An, Fan Mao, Xiandi Zhu, Guoqun Mao

**Affiliations:** 1 Department of Radiology, Tongde Hospital of Zhejiang Province, Hangzhou, 310012 Zhejiang Province China; 2 Department of Gastroenterology, Tongde Hospital of Zhejiang Province, Hangzhou, 310012 Zhejiang Province China

**Keywords:** Irritable bowel syndrome, Functional, rsfMRI, Fractional amplitude of low-frequency fluctuations, X-ray computed

## Abstract

**Background:**

The aim of the present study was to explore the brain active characteristics of patients with irritable bowel syndrome with diarrhea (IBS-D) using resting-state functional magnetic resonance imaging technology.

**Methods:**

Thirteen IBS-D patients and fourteen healthy controls (HC) were enrolled. All subjects underwent head MRI examination during resting state. A voxel-based analysis of fractional amplitude of low frequency fluctuation (fALFF) maps between IBS-D and HC was performed using a two-sample *t*-test. The relationship between the fALFF values in abnormal brain regions and the scores of Symptom Severity Scale (IBS-SSS) were analyzed using Pearson correlation analysis.

**Results:**

Compared with HC, IBS-D patients had lower fALFF values in the left medial superior frontal gyrus and higher fALFF values in the left hippocampus and right precuneus. There was a positive correlation between the duration scores of IBS-SSS and fALFF values in the right precuneus.

**Conclusion:**

The altered fALFF values in the medial superior frontal gyri, left hippocampus and right precuneus revealed changes of intrinsic neuronal activity, further revealing the abnormality of gut-brain axis of IBS-D.

## Background

Irritable bowel syndrome (IBS) is one of the most common functional gastrointestinal (GI) disorders affecting up to 11.5% of the general global population [1]. According to Rome III criteria, IBS was divided into three clinical subtypes: IBS with diarrhea (IBS-D), IBS with constipation (IBS-C), and IBS with a mixed bowel pattern (IBS-M). IBS-D is the most common subtype of IBS and has a lower disease-specific quality of life than do the other two subtypes [2]. Current treatment options for IBS-D are limited [3–5].

Though the pathophysiology of IBS is not well understood, the interaction of psychosocial factors and gut physiology has been unanimously accepted views [6–9]. The first reason is that psychosocial factors are involved in the onset of IBS and closely related to clinical efficacy [10, 11]. Studies have shown that anxiety and depression can double the risk of IBS onset [12], also aggravate gastrointestinal symptoms of IBS [13]. Another powerful reason is the reciprocal communication between central nervous system (CNS) and enteric nervous system (ENS), described as brain-gut axis [14, 15]. A review showed that several brain regions involving prefrontal lobe and cingulate gyrus, which were responsible for the process of “top-down” emotional and cognitive pain modulation, were involved in symptoms of abdominal pain of IBS [16]. Recently, one studies by Peter et al. [17] demonstrated that psychological state can change the proportion of intestinal microbiota, Daulatzai and Labus also found pathogenic gut microbiota-related systemic inflammation contributes to dysfunctional changes in brain regions including the hippocampus, underscoring the role of brain-gut-microbiota interactions in IBS. [18, 19].

Several lines of evidence from brain imaging studies have shown that IBS is closely related to structural and functional changes of brain [20, 21], further suggesting the important role for gut-brain-axis (GBA) in the pathogenesis of IBS. Using MRI and voxel-based morphometry method, Seminowicz et al. [22] examined brain anatomical differences between IBS patients and healthy individuals. They further analyzed subgroups and subclinical symptoms, and found that IBS-D was associated with changes of the posterior parietal cortex (PPC)/middle frontal gyrus (MFG)/bilateral ventral striatum/pregenual anterior cingulate cortex (ACC), pain symptoms with MFG, ventral striatum, ventrolateral prefrontal cortex (vlPFC), Orbitofrontal Cortex (OFC), emotional symptom with MFG, hippocampus and thalamus[22]. Based on functional MRI, Guleria et al. [23] examined brain responses to rectal balloon distension. They reported that the inferior orbito-frontal cortex, left calcarine, and bilateral fusiform gyri were activated in IBS-D and that the right mid-cingulate cortex was activated in IBS-C [23]. These neuroimaging studies showed that brain networks, including cognitive and emotional networks, were involved in pathogenesis of IBS, and there was heterogeneity among different subgroups.

Resting-state fMRI is a more important tool to examine brain functional activities of IBS when intestinal tract is at rest state. Based on the amplitude of low-frequency fluctuation (ALFF) and rsfMRI, Ma et al. [24] applied rsfMRI and the amplitude of low-frequency fluctuation (ALFF) method in IBS patients and found that the left superior frontal gyrus, right MFG, right hippocampus, right superior temporal pole, and bilateral postcentral gyrus exhibited lower ALFF values, while the left calcarine and left median cingulate exhibited higher ALFF values. There was a significant correlation between duration of disease in IBS and ALFF values in the altered regions [24]. Qi et al. [25] reported that IBS patients had decreased ALFF values in several core default mode network regions and increased ALFF values in the bilateral posterior insula and cuneus.

However, the brain function of IBS-D patients during resting-state is still unclear. Therefore, we chose IBS-D patients as research subjects. All subjects underwent rsfMRI. fALFF was calculated to analyze the rsfMRI data. Considering the role of psychosocial factors in IBS [26], we hypothesized that IBS-D patients have abnormal activity in emotional and cognitive areas.

## Methods

### Subjects

Twenty-seven right-handed subjects were recruited from Tongde Hospital of Zhejiang Province, including 13 IBS-D patients (8 men, 5 women; mean age, 32.23 ± 5.96 years; range 24–40 years) and 14 healthy controls (HCs, 8 men, 6 women; mean age, 29.14 ± 5.92 years; range 24–44 years). The mean duration of IBS was 19.31 ± 4.50 months (range 12–24 months). The diagnostic criteria used for IBS-D were the Rome III criteria [27]. Exclusion criteria included pregnancy, substance abuse, abdominal surgery, tobacco dependence and psychiatric illness. In addition, IBS-D subjects with current, regular use of analgesic drugs were also excluded. The IBS Symptom Severity Scale (IBS-SSS) was completed before scanning to determine IBS severity [28]. All procedures were approved by the ethics committee of ×  ×  × Hospital, and all subjects provided informed consent.

### MRI data acquisition

All experiments were performed with a 3T MRI scanner (Siemens Magnetom Verio, Erlangen, Germany) with an 8-channel birdcage head coil and foam padding to lessen motion artifact. All subjects (include HCs) were instructed to close their eyes and keep still, and not to think of anything systematically. rsfMRI images were collected by using an echo-planar imaging sequence (TE = 30 ms, TR = 2000 ms, FOV = 240 × 240 mm^2^, in-plane resolution = 64 × 64, flip angle = 90°, 36 axial slices). A total of 200 brain volumes were acquired by each acquisition. A 3D high-resolution T1-weighted anatomic image was collected by using magnetization-prepared rapid gradient echo images (TE = 2.48 ms, TR = 1900 ms, in-plane resolution = 256 × 256, TI = 900 ms, flip angle = 9°, FOV = 240 × 240 mm^2^, 76 sagittal slices, slice thickness/gap = 1/0 mm).

### Functional data preprocessing

Functional data analysis was performed using Data Processing Assistant for rsfMRI (DPARSF) programs [29] ground on the rsfMRI data analysis toolkits (REST) and statistical parametric mapping (SPM8.0). A total of 200 volumes were scanned and the first 10 volumes were discarded in order to equilibrate the initial magnetic resonance signal and adapt the subjects to the conditions. The remaining 190 consecutive volumes were used for analysis. Subsequently, the following procedures were performed: slice-timing adjustment, head-motion correction, spatial normalization to the Montreal Neurological Institute (MNI) template (resampling voxel size = 3 mm × 3 mm × 3 mm), smoothing with an isotropic Gaussian kernel (6 mm), detrending and filtering (0.01–0.08 Hz). Any subjects with a head motion greater than 2 mm translation or a 2.0° rotation in any direction were excluded.

### fALFF calculation

fALFF was analysed using REST software (http://www.restfmri.net). The preprocessed time series data were first changed into a frequency domain with a fast Fourier transform, and thus the power spectrum was obtained. We calculated square root of the power spectrum at each frequency and then acquired the mean square root across 0.01–0.08 Hz at each voxel. Finally, fALFF was computed as the ratio of the value of low frequency power spectrum (0.01–0.08 Hz) to the power spectrum of the entire frequency range.

### Statistical analysis

A second-level, random-effect, two-sample t-test was performed on the individual normalized fALFF maps on a per-voxel basis to compare the differences in fALFF between the groups. A Monte Carlo clusterwise simulation program-3DClustSim (http://afni.nimh.nih.gov) was used to correct false discovery rate. A *p* value < 0.005 with a cluster size of > 28 voxels, corresponding to a corrected *p* value < 0.05, was considered statistically significant. All coordinates were reported in Montreal Neurological Institute coordinates, which used by SPM.

### Correlation analysis

To investigate the association between the fALFF values and clinical variables, disease duration and severity, a Pearson correlation analysis was performed between the *Z* value of the abnormal brain regions and the disease duration and IBS-SSS scores of IBS-D patients in a voxelwise manner. The statistical threshold was set at *p* < 0.05 (after FDR correction).

## Results

### Demographic and clinical characteristics

There were no significant differences between the IBS-D and healthy control (HC) groups in terms of age, sex distribution or level of education. IBS-D patients had higher scores on the IBS-SSS (Table 1).

**Table 1:** Demographic and clinical characteristics

	IBS-D	HCs	*t*/*x*^2^	*p*
Sex, *n* (M/F)	13(8/5)	14(8/6)	0.054	0.816
Age, years	32.23 ± 5.96	29.14 ± 5.92	1.350	0.189
Education, years	15.69 ± 0.85	16.14 ± 0.83	1.388	0.177
Course (months)	16.6 ± 5.10	/	/	/
IBS-SSS	225.8 ± 47.4	0	/	/

Data are presented as the mean ± standard deviation. Independent-samples *t*-tests and *x*^2^ statistics were used for data analysis

*IBS-D* Diarrhea-predominant irritable bowel syndrome, *HCs* healthy controls, *M* MALE, *F* female

### Differences in fALFF between the two groups

**Fig. 1:**
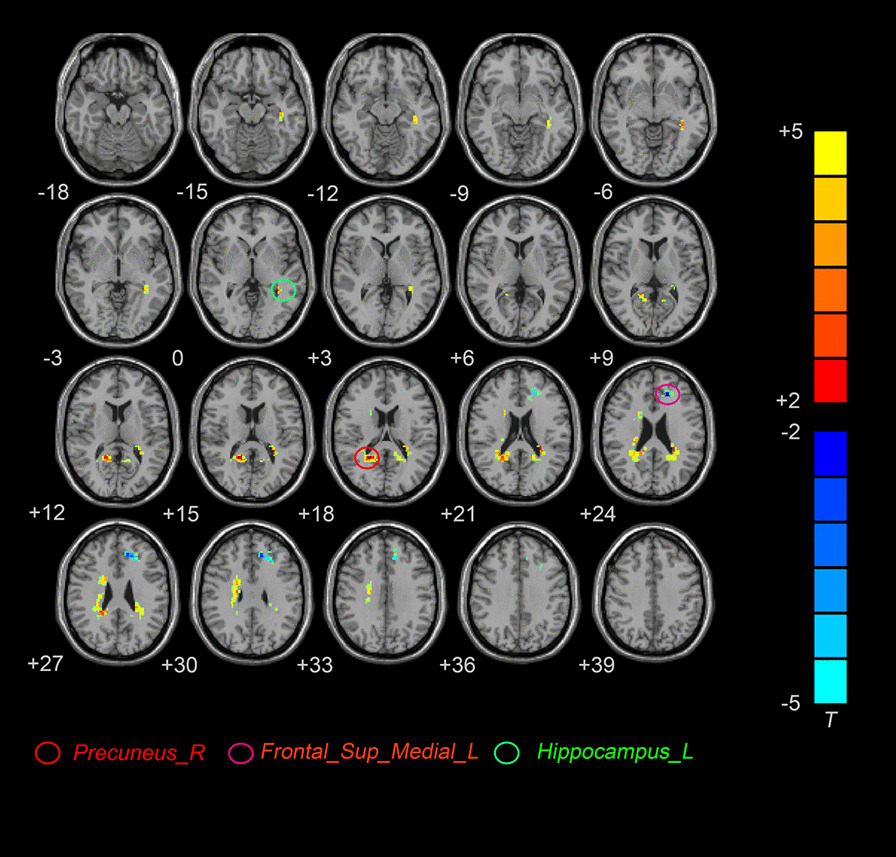
Axial map of brain regions showing higher fALFF (red) and lower fALFF values (blue) in the D-IBS group compared with the HC group (*P* < 0.005, AlphaSim corrected). The T-value scale is to the right of the image

Compared with HCs, IBS-D patients had lower fALFF in the left medial superior frontal gyri, and they had higher fALFF in the left hippocampus and right precuneus (Table 2, Fig. 1).

**Table 2:** Brain regions with different fALFF values between the two groups

Brain regions *x y z*	Voxels	BA	MNI coordinates			*T* value
*IBS-D > HCs*						
Left hippocampus	42	20	− 36	− 39	0	5.6806
Right precuneus	36		21	− 48	18	6.7449
*IBS-D < HCs*						
Left medial superior frontal gyri	70	32	− 18	39	24	− 5.8257

*IBS-D* IBS with diarrhea, *HCs* healthy controls, *BA *Brodman area

### Correlations between abnormal fALFF and clinical variables in the patients

**Fig. 2:**
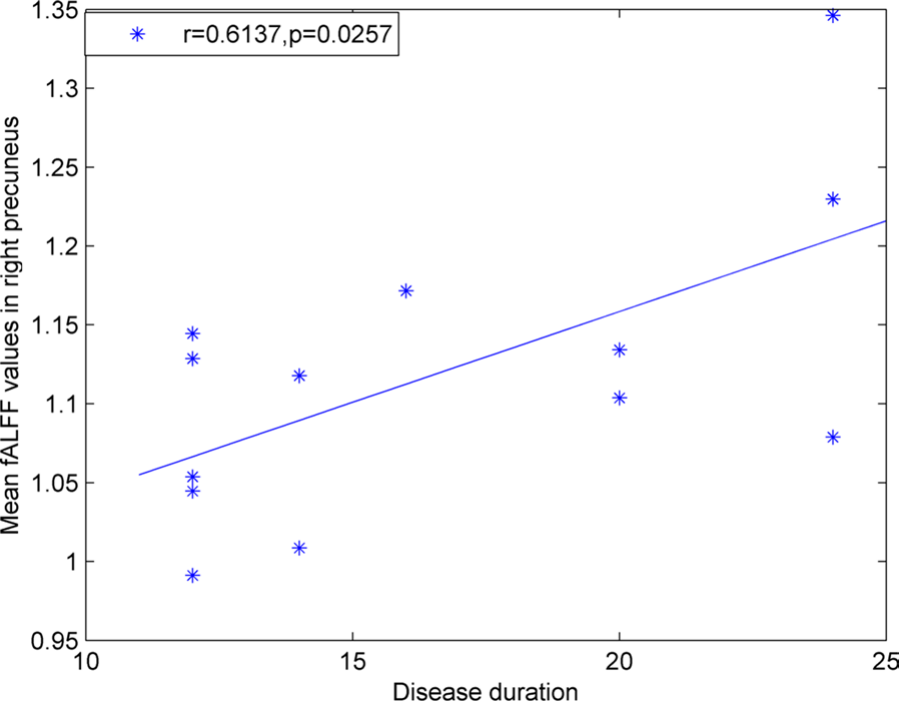
Pearson correlation analysis between D-IBS disease duration and the fALFF value in the right precuneus

We found a positive correlation between the duration of IBS-D and the fALFF value in the right precuneus (*r* = 0.6137, *p* = 0.0257). No correlations were found between IBS-SSS scores and fALFF values in other two brain regions (Fig. 2).

## Discussion

IBS is heterogeneous, not only because of the clinical phenotypes but also because of the possible pathogeneses. IBS-D is an important subtype of IBS characterized by visceral hypersensitivity and closely related to psychological states [30]. Therefore, the regulation of GBA plays an important role in IBS-D. In the current study, we used fMRI and fALFF methods to examine spontaneous neural activity in IBS-D patients during resting state. Our results demonstrated that IBS-D patients had lower fALFF in the left medial superior frontal gyri, and they had higher fALFF in the left hippocampus and right precuneus than in the HCs. Additionally, there was a positive correlation between the duration of IBS-D and the fALFF value in the right precuneus. To the best of our knowledge, this study was the first to examine the functional activity of neurons in IBS-D patients at rest, further confirming the important role of the central nervous system (CNS) in the pathogenesis of IBS-D.

IBS-D patients showed lower activity in the left medial superior frontal gyri than in the HC. There may be several reasons for this observation. First, the hypothalamic–pituitary–adrenal (HPA) axis forms a key component of GBA, and a large number of studies have confirmed that the HPA is overactive in IBS patients, giving rise to abnormalities in the enteric nervous system (ENS) [15, 31]. It is widely known that hypothalamic communication with the cerebral cortex is crucial for a wide variety of psychological and physiological functions, including maintaining neuroendocrine circadian rhythms and managing affective processes. There is a structural and functional connection between the prefrontal cortex (PFC) and the hypothalamus that regulating emotional responses to the environment; these are called top-down cognitive control mechanisms [32–34]. Recent research suggests that reappraisal, a top-down strategy of emotional regulation, is more effective in decreasing self-reported adverse effect when emotions are generated top-down versus bottom-up [35, 36]. Previous studies have shown that IBS patients had structural and functional changes in the PFC. Andresen et al. [37]. Found that IBS patients responded with lower activations of the PFC to both subliminal and supraliminal stimulation relative to controls, suggesting disturbances in the associative and emotional processing of visceral sensations. Based on rsfMRI and ALFF methods, Qi et al. [25] and Ma et al. [24] also reported that IBS patients had lower ALFF in the medial prefrontal cortex, middle frontal cortex, right orbital part of the superior frontal gyrus and anterior cingulate cortex than that of healthy controls. Therefore, the lower activity in the frontal lobe in our study was consistent with previous research results, indicating that the inhibitory effect of the frontal lobes on the HPA was attenuated. It also indicates maladjustment of emotional cognitive control in IBS patients. Second, the frontal lobe is an integral part of the central descending pain control system [38]. Previous reports have revealed that the IBS group had lower reliable activation primarily in cortical regions involved in modulation of attention as well as pain, including the lateral/medial prefrontal cortex, supramarginal gyrus (BA 40) and hippocampus [39–41]. Coen et al. [42] found that prefrontal area activity under visceral pain conditions was reduced in a working memory task acting as a distractor in comparison with no distractor. Therefore, the decreased activity in the frontal lobe in our study may reflect insufficient pain-modulating capacity in IBS-D patients.

Our study shows that IBS-D patients had enhanced functional activities in hippocampus and precuneus, in line with previous studies based on immunohistochemistry, fMRI and structural MRI [22, 37, 43]. Both the hippocampus and precuneus are important nodes of two brain networks. One is default mode network (DMN), that plays a key role in internally directed or self-generated thought, known as cognitive processes [44]. Another is Papez circuit, that is associated with processing of memory and emotion [45]. Several lines of research have shown that early-life stress is a key risk factor for IBS [46, 47], and a few researchers have explored the effect of early stress on the CNS. Graham et al. [48] reported that infants who exposed to interparental conflict showed connective changes between DMN regions. Sripada et al. [49] also reported that childhood poverty was not only associated with reduced DMN connectivity but also with higher cortisol levels in anticipation of social stress. They considered that the alterations in the DMN may be associated with less efficient cognitive processing or greater risk for development of stress-related psychopathology. The study by Chen et al. [50] demonstrated that the IBS model induced by neonatal maternal separation enhanced the expression of GluR2 and facilitated LTP in the hippocampus, possibly leading to the formation of visceral hypersensitivity at older ages. Therefore, unpredictable, stress-provoking early-life experiences may influence adolescent cognitive and emotional outcomes by disrupting the maturation of the underlying brain networks [51]. An interesting and important feature for IBS patients is that auditory stress, in the absence of direct stimulation of the rectum, can induce symptoms of IBS, further confirming the role of the CNS in IBS [37, 52, 53]. Therefore, we have reason to conclude that IBS patients integrate external stimulation (such as auditory stress) with internal experience (from adverse early-life experiences stored in the hippocampus that may be unconscious) and then appraise using the cognitive network, and cognitive bias and emotional dysregulation will be produced due to the altered networks and affect the enteric nervous system (ENS). Our results indicating increased functional activity in the hippocampus and precuneus may suggest that IBS-D patients have amplified sensitivity to external stimuli.

The last important result in our study was the positive correlation between disease duration and the fALFF value in the right precuneus. The changes in enteric microbiota due to disease course may be one reason, just as the study by Malinen et al. [54] showed that IBS patients had quantitative alterations in GI microbiota after 3 months using real-time PCR assays. Lo Presti et al. [55] reported that the microbiota richness was characterized by a microbial diversity reduction in fecal and mucosal samples, going from CTRLs to IBS, and then to inflammatory bowel diseases (IBD). Putignani et al. [56] showed that the microbiota dysbiosis can be a risk factor of IBD along the childhood-adulthood transition. These changes in internal environments had a direct impact on the CNS through GBA [15, 57].

Another important reason may be chronic stress. IBS was confirmed to be a long-lasting, recurring disorder. On average, IBS patients have symptoms for 7 days in a month, and the average number of bouts on an affected day was two, with each bout lasting an hour [58]. Lembo et al. [59] reported that IBS patients with longstanding disease (> 5 years) had significantly increased phobia scores, anxiety scores, paranoia scores, and hostility scores on the SCL-90 scale compared to those with recent onset (> 2 years), suggesting IBS patients with long symptom duration had more psychological symptoms. Therefore, the disease itself and the duration are chronic stressors. A survey also showed that the indirect and direct costs were important stressors for IBS patients [60, 61]. Those chronic stressors may act on the CNS in two ways. One way is that the stressors directly affect the CNS, as has been shown in previous studies of posttraumatic stress disorder [62], social anxiety disorder [63] and unipolar major depression [64]. Veer et al. [65] also reported that psychosocial stress can increase amygdala functional connectivity with the precuneus in healthy subjects. Another way is an indirect effect: First, stressors have major effects on gut physiology, including visceral hypersensitivity, increased permeability and changes in gastrointestinal secretion and colonic mucosa [66, 67], and then these changes in the bowel affect the CNS.

Several limitations are worth mentioning. First, the number of participants in our study was relatively small, possibly affecting the statistical power. Second, the range of disease duration was between 12 and 24 months. Whether the results of correlations between disease duration and fALFF value in the right precuneus can be applied to other ranges of disease duration should be studied further. Third, due to a lack of available scales, we could not analyze the effect of psychological factors on brain function in IBS-D patients. In future research, first, we may need to increase sample size and take measures strictly following standard procedures to avoid various factors in the surrounding environment that interfere with brain activity. Second, more studies that extending the research period and focusing on brain regions related to cognition, stress and pain need to be included. In this study, we found that specific regions of the brain were spontaneously activated in IBS-D, which may help understand central mechanism of IBS and offer reference for the future study of brain function in IBS. Finally, brain functional changes in IBS and its correlation with intestinal microflora alterations and psychological factors are worthy further studies and tests.

## Conclusion

In conclusion, we found that IBS-D patients had decreased spontaneous neuronal activity in the left medial superior frontal gyri with increased regional brain activity in the right precuneus and left hippocampus. These regions are cognitive and stress pain modulatory brain areas. Therefore, our results could be related to cognitive impairment and weak stress/pain regulation in long-term visceral sensory abnormalities.

## Data Availability

The datasets used and/or analyzed during the current study are available from the corresponding author on reasonable request.

## Abbreviations

ALFF: Amplitude of low-frequency fluctuation
DMN: Default mode network
ENS: Enteric nervous system
HPA: Hypothalamic–pituitary–adrenal
HC: Healthy controls
fALFF: Fractional amplitude of low frequency fluctuation
IBS-D: Irritable bowel syndrome with diarrhea
IBS: Irritable bowel syndrome
IBS-SSS: Irritable bowel syndrome with Symptom Severity Scale
PFC: Prefrontal cortex
rsfMRI: Esting-state funcational magnetic resonance imaging
